# Ezrin Is Highly Expressed in Early Thymocytes, but Dispensable for T Cell Development in Mice

**DOI:** 10.1371/journal.pone.0012404

**Published:** 2010-08-27

**Authors:** Meredith H. Shaffer, Yanping Huang, Evann Corbo, Gregory F. Wu, Marielena Velez, John K. Choi, Ichiko Saotome, Judy L. Cannon, Andrea I. McClatchey, Anne I. Sperling, Jonathan S. Maltzman, Paula M. Oliver, Avinash Bhandoola, Terri M. Laufer, Janis K. Burkhardt

**Affiliations:** 1 Department of Pathology and Laboratory Medicine, Children's Hospital of Philadelphia and University of Pennsylvania School of Medicine, Philadelphia, Pennsylvania, United States of America; 2 Department of Pathology and Laboratory Medicine, University of Pennsylvania School of Medicine, Philadelphia, Pennsylvania, United States of America; 3 Department of Medicine, University of Pennsylvania School of Medicine, Philadelphia, Pennsylvania, United States of America; 4 Department of Neurology, University of Pennsylvania School of Medicine, Philadelphia, Pennsylvania, United States of America; 5 Department of Pathology, Massachusetts General Hospital (MGH) Cancer Center and Harvard Medical School, Charlestown, Massachusetts, United States of America; 6 Department of Medicine, University of Chicago, Chicago, Illinois, United States of America; New York University, United States of America

## Abstract

**Background:**

Ezrin/radixin/moesin (ERM) proteins are highly homologous proteins that function to link cargo molecules to the actin cytoskeleton. Ezrin and moesin are both expressed in mature lymphocytes, where they play overlapping roles in cell signaling and polarity, but their role in lymphoid development has not been explored.

**Methodology/Principal Findings:**

We characterized ERM protein expression in lymphoid tissues and analyzed the requirement for ezrin expression in lymphoid development. In wildtype mice, we found that most cells in the spleen and thymus express both ezrin and moesin, but little radixin. ERM protein expression in the thymus was differentially regulated, such that ezrin expression was highest in immature thymocytes and diminished during T cell development. In contrast, moesin expression was low in early thymocytes and upregulated during T cell development. Mice bearing a germline deletion of ezrin exhibited profound defects in the size and cellularity of the spleen and thymus, abnormal thymic architecture, diminished hematopoiesis, and increased proportions of granulocytic precursors. Further analysis using fetal liver chimeras and thymic transplants showed that ezrin expression is dispensable in hematopoietic and stromal lineages, and that most of the defects in lymphoid development in ezrin^−/−^ mice likely arise as a consequence of nutritional stress.

**Conclusions/Significance:**

We conclude that despite high expression in lymphoid precursor cells, ezrin is dispensable for lymphoid development, most likely due to redundancy with moesin.

## Introduction

The ERM proteins ezrin, radixin and moesin tether transmembrane and cytoplasmic molecules to actin filaments at the cell cortex in a regulated manner. One or more of these three closely related proteins is expressed in every cell type examined [1], and they are known to play an important role in organizing specialized domains at the cell surface. For example, in epithelial cells, these proteins are important for organizing the apical membrane domain and its associated junctional complexes [2]–[4]. In T cells, ERM proteins are important for numerous functions including maintenance of microvilli, organization of proximal-distal T cell polarity, regulation of CD95-induced cell death, and promotion of TCR-induced signaling events leading to cytokine production [5]–[13].

Because of their high sequence similarity, ezrin, radixin, and moesin are usually thought to be functionally redundant. This idea has been borne out by the analysis of mice bearing germline deletion of individual ERM proteins, where abnormalities are largely restricted to tissues expressing only one family member. Moesin-deficient mice were initially described as phenotypically normal [14], but it was later found that these mice exhibit defects in hepatic stellate cells, in which moesin is the dominant ERM protein expressed [15]. Similarly, radixin is the dominant ERM protein expressed in the liver, and radixin-deficient mice exhibit liver defects leading to hyperbilirubinemia [16]. Ezrin-deficient mice are born in sub-Mendelian numbers; although they appear normal at birth, they runt and ultimately die within 7–10 days [4]. The lethality is thought to be due to defects in the apical terminal web of the gut epithelium, a tissue that expresses only ezrin. In addition, ezrin-deficient mice have reduced apical microvilli and basal infoldings in retinal epithelial (RPE) cells, another cell type that expresses only ezrin [17]. Therefore, as in mice deficient for either moesin or radixin, the most dramatic phenotypes of ezrin-deficient mice are revealed in tissues where a single ERM protein is expressed.

Although the phenotypes of mice lacking individual ERM proteins point to a high degree of functional redundancy, biochemical differences suggest that there may be important functional distinctions among these proteins. For example, two known tyrosine phosphorylation sites in ezrin are not conserved in moesin or radixin, and there are differences in protease sensitivity and cargo binding [9], [18], [19]. In T cells, which express moesin and ezrin in a ratio of ∼3∶1, there is evidence that loss of ezrin alone perturbs cell signaling [13]. We recently tested the issue of functional redundancy in mature T cells. We found that ezrin and moesin are differentially tyrosine phosphorylated upon T cell receptor engagement and that these proteins exhibit distinct patterns of movement with respect to the immunological synapse and distal pole complex [12]. In our hands, primary T cells deficient for either ezrin or moesin alone showed modest defects in T cell function. These defects were significantly more profound in cells deficient for both ezrin and moesin [12], indicating that there is significant functional redundancy between ezrin and moesin in mature T cells.

The expression pattern of ERM proteins within lymphoid tissues has not been carefully explored, nor has the requirement for ezrin during lymphoid development. The thymus is composed of both immature lymphocytes and thymic epithelial cells (TECs). Both cell types require actin-based cytoskeletal elements such as microvilli, and TECs are known to form junctional complexes [20]. Thus, depending on the expression patterns of ERM proteins or the independent roles of these molecules, individual ERM proteins may play an important role in T cell development.

In this report, we show both ezrin and moesin are expressed in the thymus, but that ezrin levels are highest in immature DN and DP thymocytes, while moesin levels are highest in more mature SP thymocytes. Germline deletion of ezrin results in profound defects in both the spleen and thymus. Using a combination of bone marrow chimeras and thymic transplants, we show that the defects are not attributable to a requirement for ezrin expression in hematopoietic cells or thymic stroma. Instead, these defects likely arise from nutritional stress during development. Thus, while the differential expression of ezrin and moesin suggests that these proteins can serve specialized roles during development, it seems likely that functional redundancy by moesin can support thymic development in ezrin-deficient mice.

## Results

### Ezrin and moesin are differentially expressed during lymphoid development

**Figure 1:**
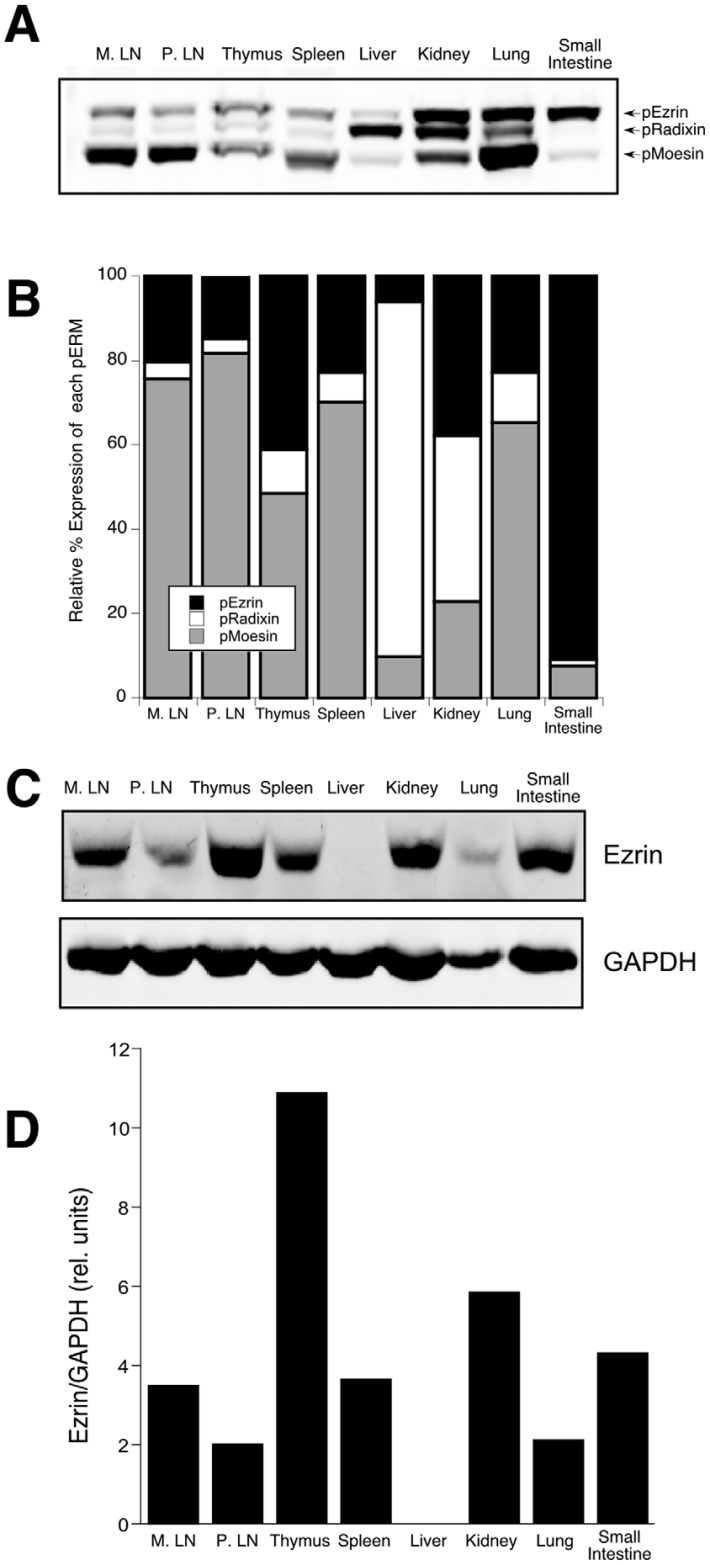
Expression of ezrin and moesin in lymphoid organs. Mouse organs were lysed in SDS sample buffer and analyzed by western blot with antibodies specific for pERM (A), and ezrin or GAPDH (C). B. Relative abundance of each pERM protein within a tissue was calculated using an Odyssey Imager and normalized to the total pERM expression in that tissue. D. Expression of ezrin among tissues was calculated after normalization to GAPDH.

In mature T cells, moesin is more abundant than ezrin, and only trace amounts of radixin are present [21]. However, little is known about the expression patterns of ERM proteins during T cell development. To assess the overall ratios of ERM protein expression, various mouse organs were analyzed by western blot. Since phosphorylation of ERM proteins on a conserved threonine is typically proportional to their relative abundance, a phospho-ERM antibody can be used to assess relative protein levels. As reported previously, small intestine expressed almost exclusively ezrin, while liver expressed mostly radixin (Figure 1A and B) [4], [22]. In lymph node and spleen, moesin accounted for ∼80% of the total pERM protein, ezrin about 20%, and radixin levels were low to undetectable. In contrast, thymus expressed a high proportion of ezrin, over 40% of the total pERM protein. Indeed, of the tissues tested, only small intestine expressed a higher proportion of ezrin.

To determine if the higher proportional expression of phospho-ezrin in the thymus translated into an increased absolute expression of ezrin, the same tissues were analyzed by western blot using antibodies specific for ezrin and normalizing to the housekeeping gene GAPDH. As shown in Figure 1 C and D, thymus expressed significantly more ezrin than any other tissue tested; total ezrin levels were 4–5 times higher in thymus than in peripheral lymphoid organs. Since the vast majority of cells in the thymus are developing T cells, these results imply that ERM expression patterns change significantly during T cell development.

**Figure 2:**
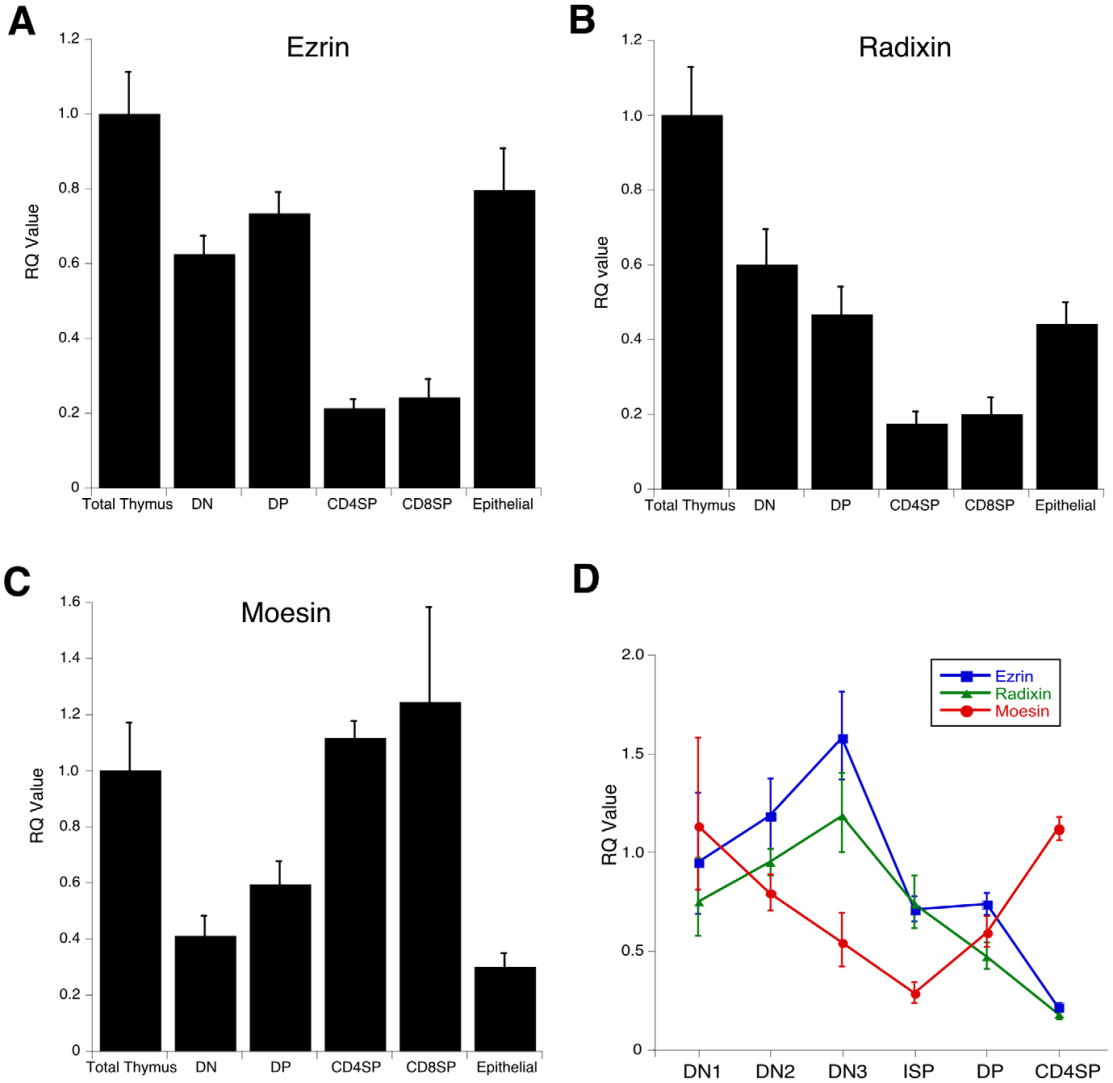
Ezrin and moesin mRNAs show reciprocal expression patterns during thymocyte development. Thymii were harvested from wildtype mice and single cell suspensions were separated on a Percoll gradient, and sorted for the populations shown. mRNA was isolated was analyzed by real-time PCR for ezrin (A), radixin (B), and moesin (C) expression, relative to total unsorted thymus. D. Expression profiles of ezrin (blue), radixin (green), and moesin (red), each normalized to its own expression in total thymus, were overlayed to facilitate comparison of relative expression patterns of ezrin and moesin. Samples were run in triplicate and are representative of 2 independent experiments. Data represent means +/− StDev.

To assess ERM expression patterns in developing thymocytes, we conducted real-time PCR analysis of sorted cell populations, normalizing each mRNA to whole thymus. As shown in Figure 2, we found that ERM mRNA expression is differentially regulated during thymic development. Expression of ezrin mRNA is high in DN and DP thymocytes, and ∼3-fold lower in CD4 and CD8 SP thymocytes (Figure 2A). The pattern of radixin mRNA expression mirrors that of ezrin (Figure 2B), but this is unlikely to be functionally significant since the absolute levels of radixin protein in the thymus are very low. Interestingly, moesin mRNA shows a reciprocal pattern, low in DN and DP thymocytes and high in CD4 and CD8 SP thymocytes (Figure 2C). Closer analysis of thymocyte subsets shows that ezrin message levels peak at the DN3 stage, drop precipitously at the DN3-DN4/ISP transition, and continue to drop as development progresses (Figure 2D). In contrast, moesin mRNA levels drop steadily during DN development, and then increase dramatically to the SP stage. Since stromal cells also contribute to ERM protein expression patterns, we also sorted thymic epithelial cells and assessed relative mRNA levels. As shown in Figure 2A–C, ezrin mRNA is expressed in TECs at 80% of the levels in the thymus as a whole, while moesin (and radixin) mRNA expression is much lower in TECs than in whole thymus. Since ezrin and moesin protein levels are roughly equal in thymus, (and assuming that expression is regulated largely at the transcriptional level) this indicates that ezrin is the predominant ERM protein expressed in TECs.

### Ezrin and moesin show distinct distributions within lymphoid organs

**Figure 3:**
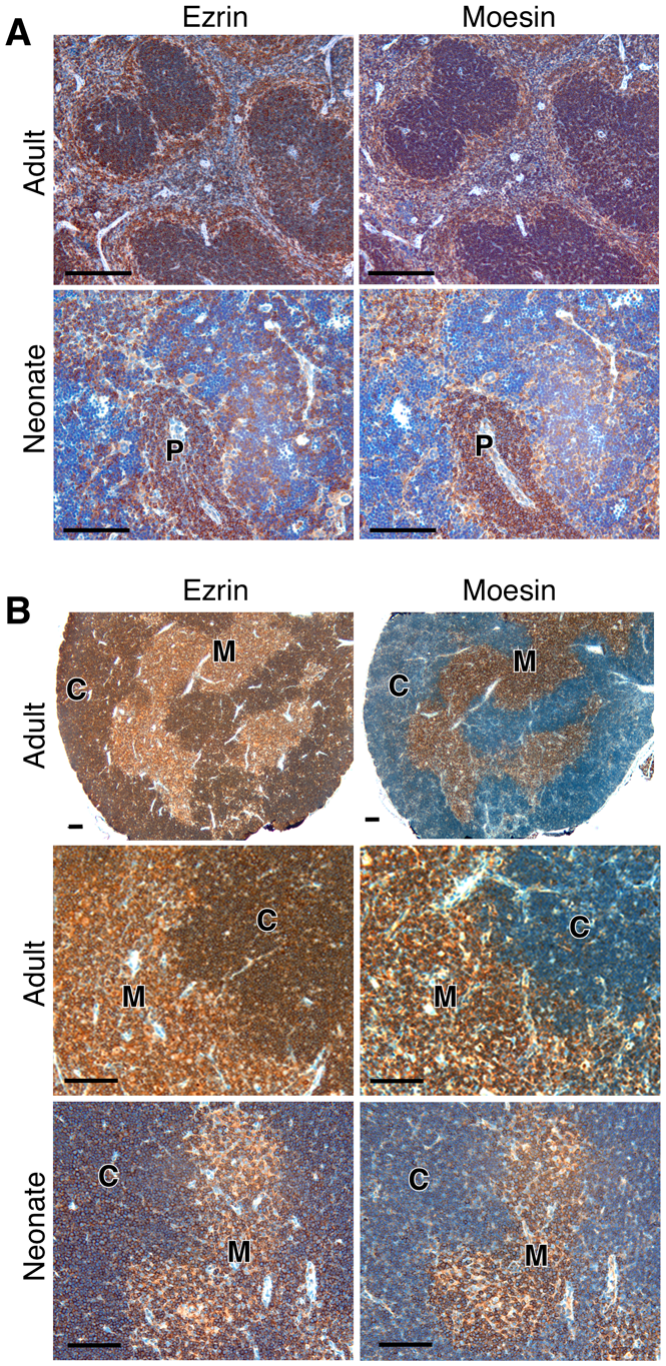
Ezrin and moesin are differentially expressed within the thymus. Spleens (A) and Thymii (B) from wildtype mice at 6–8 weeks (Adult) or P3–P5 (Neonate), were fixed, sectioned and stained using antibodies against ezrin or moesin (brown) in combination with hematoxylin staining (blue). Organ structures are labeled as thymic cortex (C), thymic medulla (M), and peri-arteriolar lymphoid sheath (P). Bars = 100uM.

As another way of exploring differential expression of ezrin and moesin, lymphoid organs from neonatal and adult mice were fixed and analyzed by immunohistochemistry. Similar reactivity for both ezrin and moesin was found throughout the spleen, with the highest levels of staining occurring in the white pulp, marginal zone, and PALS regions (Figure 3A). At higher power, most lymphocytes were positive for both ezrin and moesin, although a subset of lymphocytes appeared negative for ezrin expression. Immature erythroid cells were weakly positive for both ezrin and moesin, while mature erythroid cells, megakaryocytes, and neutrophils were negative for both proteins (data not shown). Ezrin and moesin expression patterns were similar in adult and neonatal mice. Analysis of the thymus revealed distinct patterns of ezrin and moesin expression (Figure 3B). Consistent with RNA-expression studies, we found that ezrin expression was somewhat higher in the cortex, where thymocytes progress through the DN and DP stages of development and selection takes place. However, moesin was most highly expressed in the medulla, a region enriched in more mature, SP T cells. Closer analysis showed that strong ezrin expression was detectable in nearly 100% of the thymic cortex cells, but a subset of non-lymphocytic cells in the medulla expressed low levels of ezrin (data not shown). Conversely, while some moesin reactivity also was seen in most cells in the adult cortex, cells in the medulla showed stronger reactivity. This pattern was also observed in younger mice (Figure 3B, bottom panels). In addition, ezrin appears to be present in cTECs but negative in mTECs,. Taken together, these studies suggest that ezrin may be particularly important for early stages of thymic development.

### Ezrin^−/−^ mice have a severe paucity of cells in the spleen and thymus

**Figure 4:**
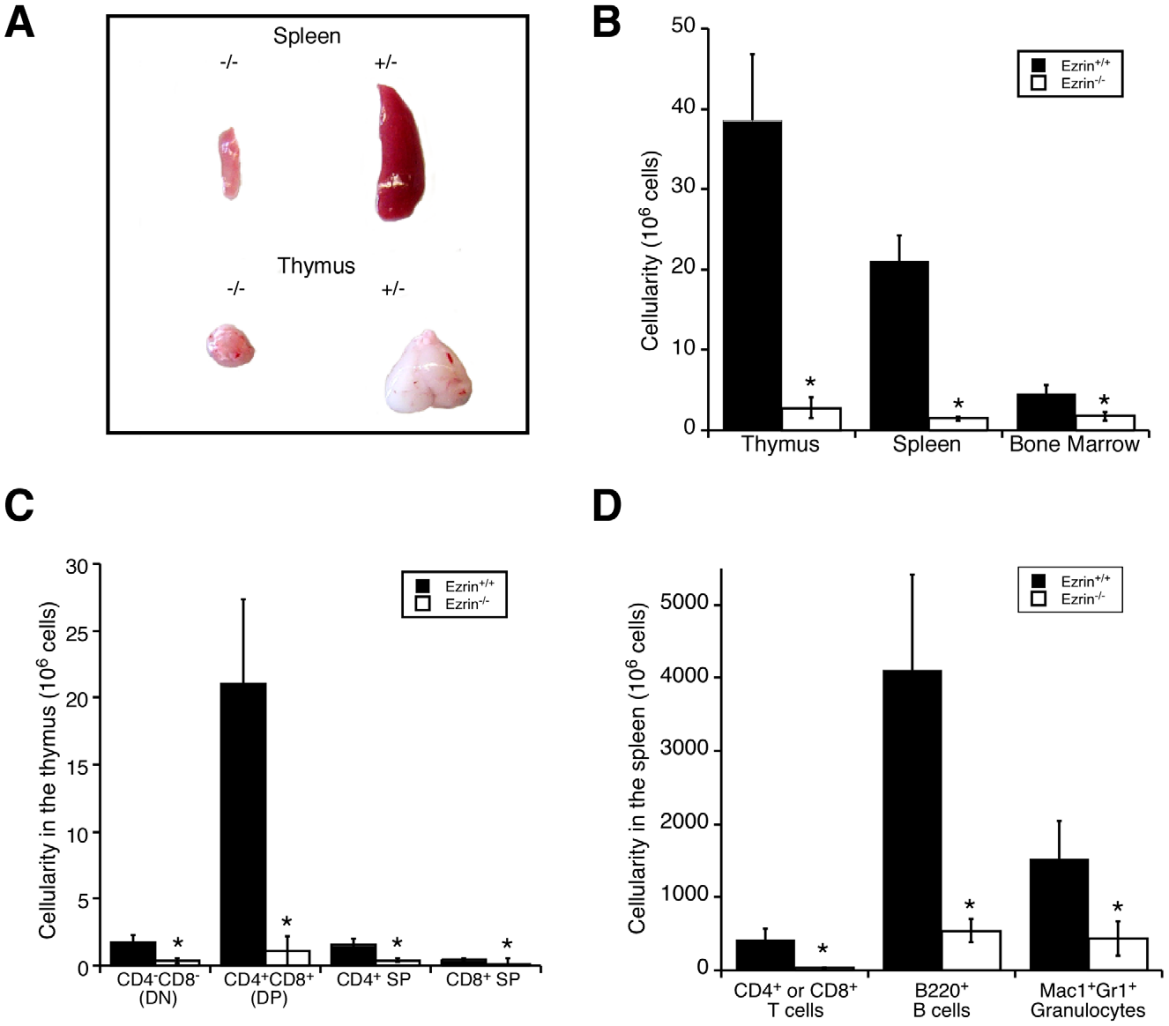
Germline deletion of ezrin results in profound defects in lymphoid development. A. Spleens and thymii from ezrin^−/−^ neonatal mice and wildtype littermates were collected and photographed. B. Single cell suspensions were counted for total cellularity. C and D. Absolute immune cell populations from the thymus (C) or spleen (D) were calculated by antibody staining followed by flow cytometry. Data shown are means +/− StDev of at least 3 mice, all at P3–P5. *p≤0.05.

To test the idea that ezrin is uniquely important for early thymic development, we analyzed mice bearing a germline deletion of the ezrin gene. As shown previously [4], ezrin^−/−^ mice were born in submendelian ratios but appeared normal at birth. Two to three days after birth, the ezrin^−/−^ mice began to runt, and they died within 7–10 days. We therefore assessed mice within seven days of birth. At this time, ezrin^−/−^ mice are runted, weighing two to three times less than wildtype littermates (data not shown). Therefore, we expected the immune organs to be proportionally smaller than wildtype littermates. Surprisingly, both the spleens and thymii from these mice were disproportionately small and showed severe reductions in cellularity (Figure 4A and 4B). The bone marrow was pale and also had decreased cellularity, but the differences were less severe than for the spleen or thymus.

**Figure 5:**
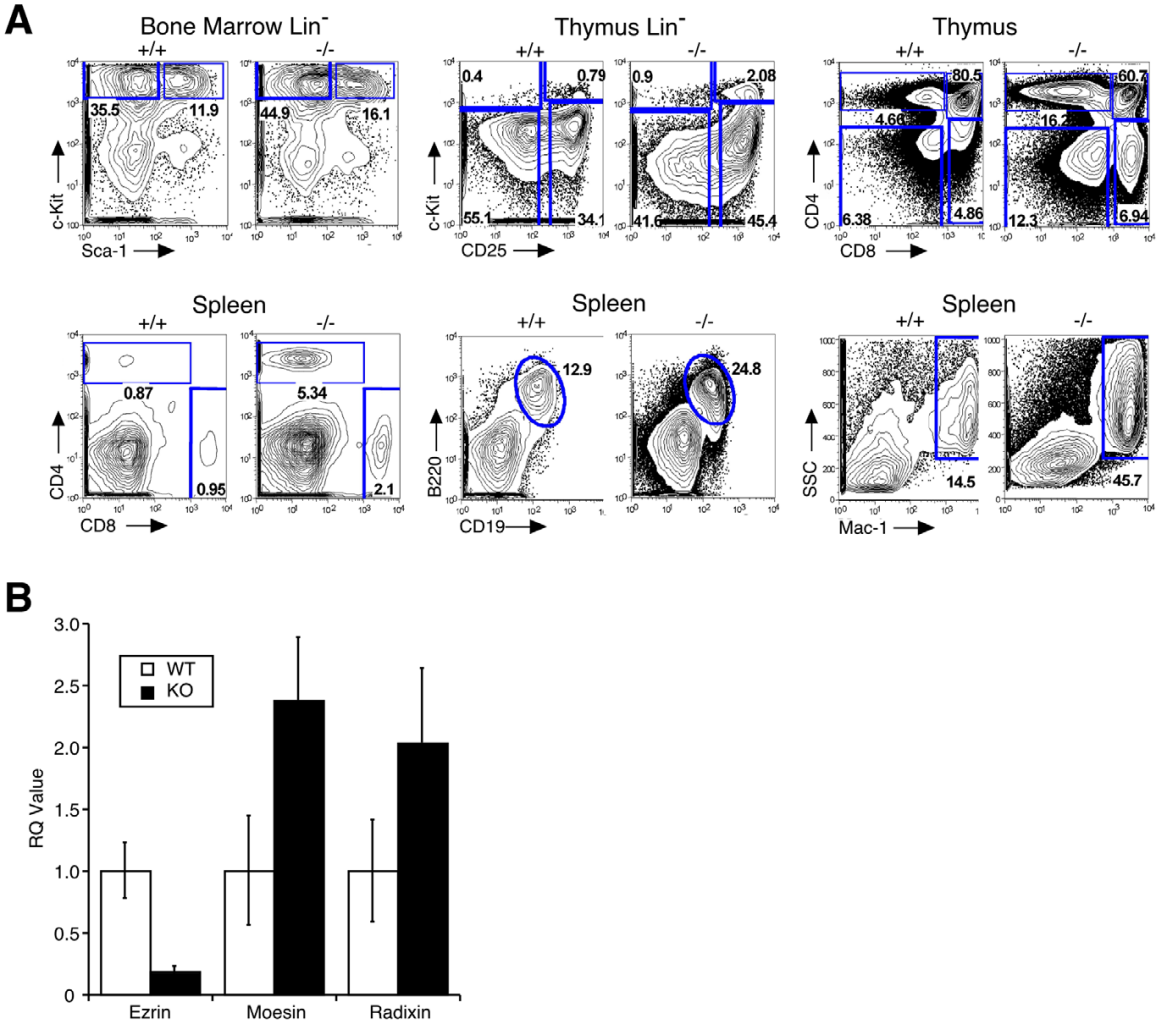
Germline deletion of ezrin results in altered lymphoid populations and upregulation of moesin and radixin mRNA. A. Single cell suspensions were made from the spleen, thymus, and bone marrow of P3–P5 ezrin^−/−^ mice and wildtype littermates. Cells were stained with indicated antibodies, and analyzed by flow cytometry. B. Thymii were harvested from ezrin^−/−^ and mice and littermate controls, and mRNA was isolated and analyzed by real-time PCR. Expression of each mRNA was normalized to control. Data represent means from 4 mice of each genotype, each run in triplicate, +/− SEM.

To ask if the decreased cellularity was due to the preferential loss of a particular cell type, lymphoid sub-populations were analyzed. As shown in Figure 4C, the total numbers of DN, DP, and SP CD4^+^ and CD8^+^ T cells in the thymus were dramatically decreased. No absolute blocks in T cell development were seen. All lineage-negative populations were represented in bone marrow and thymus, although there were modest increases in the proportion of CD25^+^ precursors, and decreases in the proportion of CD4^+^CD8^+^ DP thymocytes (Figure 5, top panels). In the spleen, population analysis showed although the total numbers of T cells, B cells, and granulocytes were decreased (Figure 4D), the relative proportion of each of these populations was increased (Figure 5, bottom panels). The increased proportions of these cells was balanced by a substantial decrease in mature erythroid cells, as determined by histology (data not shown).

To determine if the decrease in splenic erythroid populations was a reflection of altered erythrogenesis, blood samples were collected and analyzed. Surprisingly, ezrin^−/−^ mice did not appear to be anemic based on this assay. Instead, ezrin^−/−^ mice displayed an increased hematocrit, platelet and red blood cell (RBC) counts (Table 1). As discussed below, the increased hematocrit in ezrin^−/−^ mice may be due to the effects of dehydration in these mice.

**Table 1:** Ezrin^−/−^ mice have altered blood cell counts.

	Ezrin^+/+^	Ezrin^−/−^	ttest
**RBC**	4.05+/−0.23	5.41+/−0.48	*0.0008*
**Hb**	9.06+/−0.62	12.13+/−0.92	*0.0009*
**HCT**	37.04+/−2.57	52.47+/−4.68	*0.0006*
**MCHC**	24.48+/−1.09	23.19+/−0.85	*0.0162*
**RDW**	22.71+/−1.7	26.40+/−3.24	*0.0209*
**WBC**	3.81+/−1.55	3.28+/−0.95	*0.5505*
**Ly**	2.01+/−0.90	1.46+/−0.37	*0.1901*
**PLT**	389+/−120	721+/−144	*0.0036*
**MPV**	5.01+/−0.17	5.26+/−0.08	*0.0148*

Blood samples were collected from ezrin^−/−^ and wildtype littermates in EDTA-containing tubes. Analysis was done using an automated hematocrit analyzer. Shown are averages and standard deviations of 7 ezrin^−/−^ and 14 ezrin^+/+^ mice. RBC (Red Blood Cells, M/µL), Hb (Hemoglobin, g/dL), HCT (Hematocrit, %), MCHC (Mean Corpuscular Hemoglobin Concentration, g/dL), RDW (Red Cell Distribution Width), WBC (White Blood Cells, K/µL), Ly (Lymphocytes, K/µL), PLT (Platelets, K/µL), MPV (Mean Platelet Volume, fL).

### Moesin and radixin are upregulated in the thymi of ezrin^−/−^ mice

Given the defects in lymphoid populations observed in ezrin^−/−^ mice, we asked if there is compensatory upregulation of moesin and radixin. RNA was generated from thymii of ezrin^−/−^ mice and littermate controls, and analyzed by real-time PCR. As shown in Fig 5B, levels of both moesin and radixin mRNAs increased approximately 2-fold in ezrin^−/−^ mice.

### Ezrin^−/−^ mice have abnormal thymic architecture

**Figure 6:**
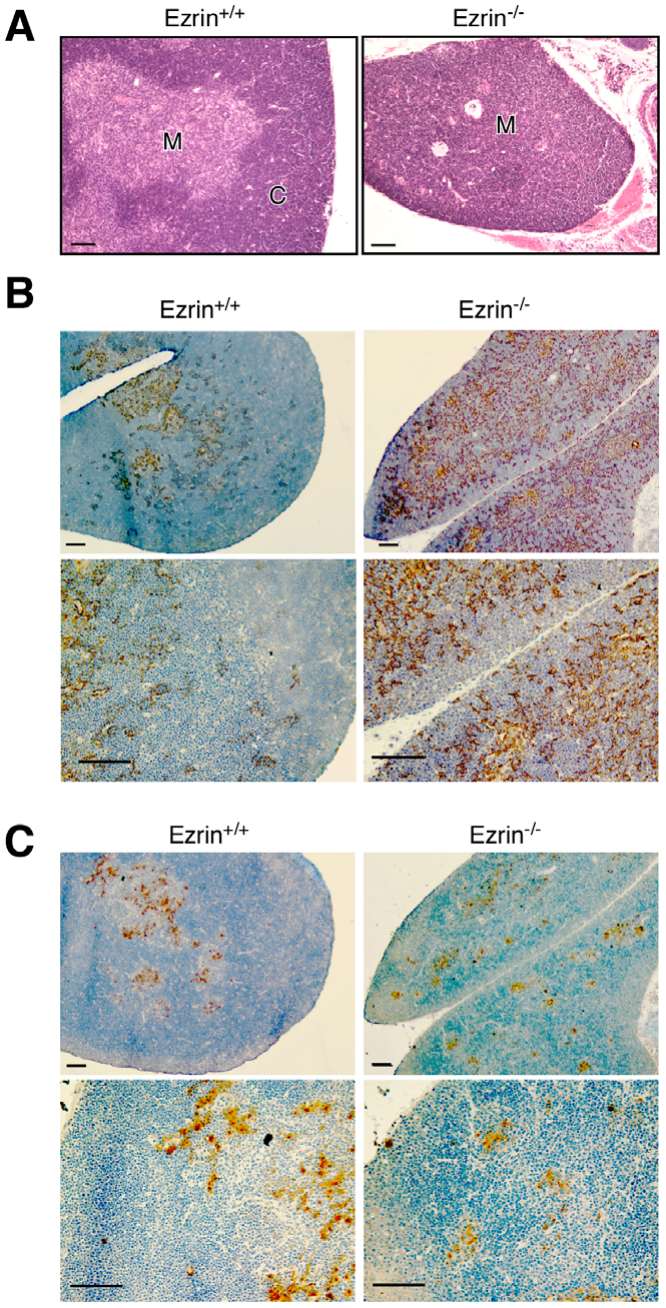
Germline deletion of ezrin results in altered thymic architecture. Thymii were dissected from ezrin^−/−^ mice and wildtype littermates at P7 and either fixed with formalin (A) or frozen (B and C). Formalin-fixed samples were analyzed by hematoxylin and eosin staining (A), and frozen samples were stained with K5-specific antibody (B) or UAE (C) in combination with hematoxylin staining. Scale bars indicate magnification. C, cortex; M, medulla. Bars = 100uM.

Crosstalk between the developing thymic stroma and bone marrow-derived thymocytes is necessary for the normal development of both. Thus, we asked if the defects in thymocyte development in ezrin^−/−^ mice are accompanied by abnormalities in thymic architecture. Based on H&E staining (Figure 6A), ezrin^+/+^ thymii had a clearly defined cortex and medulla, but ezrin^−/−^ thymii lacked a discernable cortex. To explore these architectural defects, thymic sections were stained with the medullary TEC markers, K5 and UAE (Figure 6B). Consistent with the H&E staining, WT mice showed a well-defined medullary region with both markers. In contrast, K5 labeling of thymii from ezrin^−/−^ mice showed an enlarged medullary region. Moreover, labeling for the more specific medulla marker, UAE, was markedly patchy, indicating that the structure of the medulla is also affected. Together, these data show that both the thymic cortex and medulla of ezrin^−/−^ mice are severely abnormal.

### Major lymphoid defects are independent of hematopoietic or stromal lineages, and occur after manifestation of gut defects

**Figure 7:**
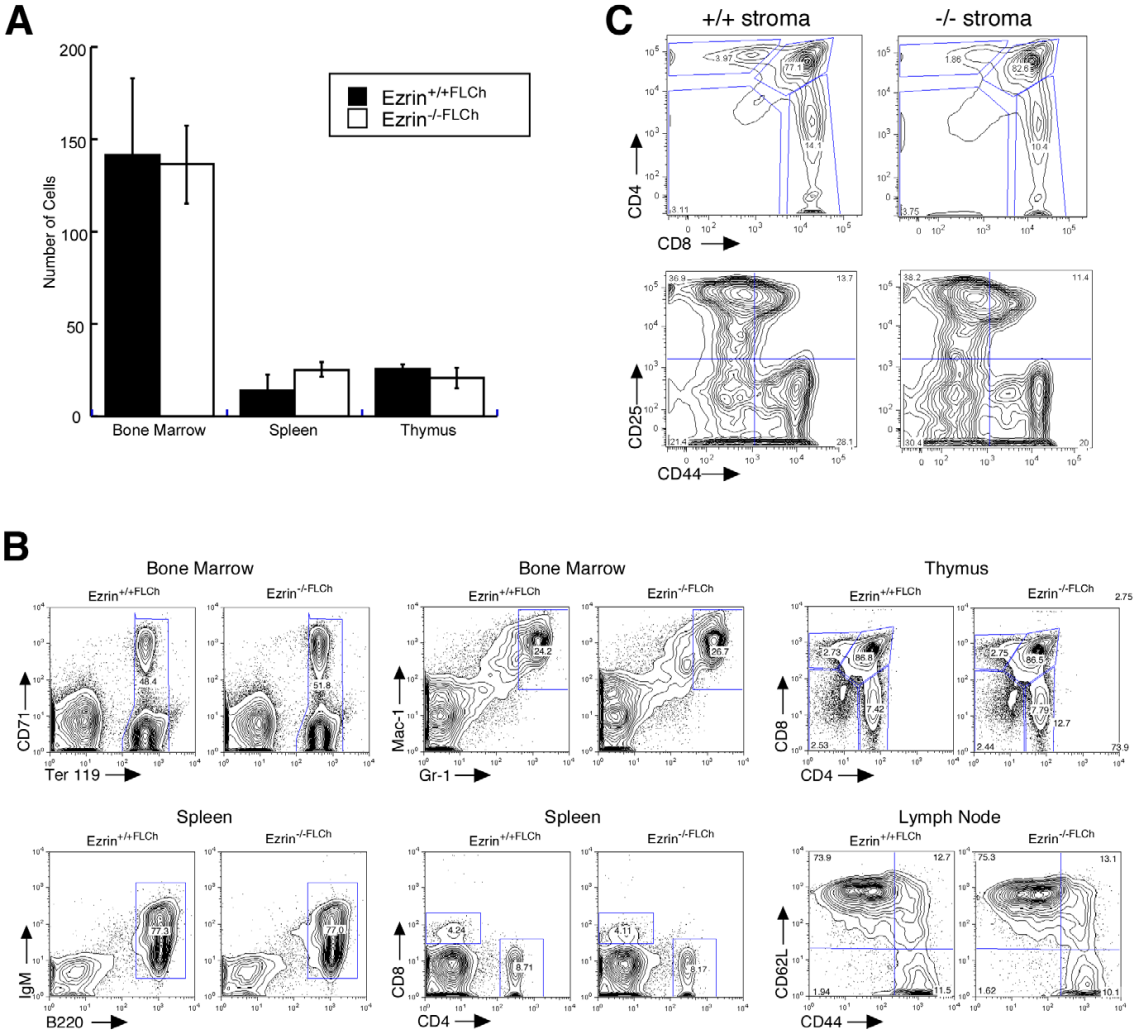
Development proceeds normally in fetal liver chimeras and transplanted thymii. A and B, Fetal liver cells isolated from E14 ezrin^−/−^ or wild type controls were injected into lethally irradiated congenic wildtype recipients. Bone marrow, spleen and thymii were harvested at 6 weeks and analyzed by flow cytometry. A, Cellularity of donor cells was assessed. B, Population analysis of CD45.2 donor cells. C, Thymii from one to three day old ezrin^−/−^ mice or littermate controls were transplanted into wildtype recipients. After nine weeks, thymii were harvested and analyzed by flow cytometry.

To ask if the defects we observe in ezrin^−/−^ mice are due to intrinsic defects in hematopoietic lineage cells, we generated chimeras by injecting fetal liver cells from ezrin^−/−^ and ezrin^+/+^ E14 embryos into lethally-irradiated wildtype congenic mice. Blood samples collected from chimeric mice six weeks post-reconstitution indicated that the loss of ezrin had no effect on erythropoiesis, since normal RBCs, hematocrit, platelets, and hemoglobin were found (Table 2). In addition, normal cellularity was seen in the spleen, thymus, and bone marrow (Figure 7A). Analysis of cell populations from these mice showed normal development of RBCs, granulocytes, B cells, and T cells (Figure 7B). Moreover, levels and frequency of TCRβ expression in the spleen, LN, and thymus were similar in wt and ezrin^−/−^ cells (data not shown). We conclude that expression of ezrin in hematopoietic-lineage cells is not required for lymphoid development.

**Table 2:** Ezrin^−/−^ fetal liver chimeras have normal blood cell counts.

	Ezrin^+/+^	Ezrin^−/−^	ttest
**RBC**	8.97+/−1.57	9.85+/−2.27	*n.s.*
**Hb**	12.25+/−2.52	12.2+/−1.43	*n.s.*
**HCT**	42.03+/−7.36	47.93+/−13.63	*n.s.*
**MCHC**	29.20+/−3.98	26.53+/−5.06	*n.s.*
**RDW**	19.07+/−1.61	20.20+/−1.85	*n.s.*
**WBC**	3.53+/−1.49	8.22+/−6.78	*n.s.*
**Ly**	2.64+/−1.19	6.69+/−6.23	*n.s.*
**PLT**	281+/−224	486+/−249	*n.s.*
**MPV**	5.70+/−.33	5.9+/−0.95	*n.s.*

Blood samples were collected from ezrin^−/−^ and ezrin^+/+^ fetal liver chimeras in EDTA-containing tubes. Analysis was done using an automated hematocrit analyzer. Shown are averages and standard deviations of 4 ezrin^−/−^ and 14 ezrin^+/−^ or 6 ezrin^+/+^ chimeric mice. RBC (Red Blood Cells, M/µL), Hb (Hemoglobin, g/dL), HCT (Hematocrit, %), MCHC (Mean Corpuscular Hemoglobin Concentration, g/dL), RDW (Red Cell Distribution Width), WBC (White Blood Cells, K/µL), Ly (Lymphocytes, K/µL), PLT (Platelets, K/µL), MPV (Mean Platelet Volume, fL).

To assess the requirement for ezrin expression in thymic stromal cells, thymii from WT or ezrin^−/−^ mice were implanted subcutaneously into WT recipients and allowed to repopulate with WT thymocytes. After nine weeks, thymii were harvested and thymocytes were analyzed by flow cytometry. Though the size of the transplanted thymii varied substantially from mouse to mouse, ezrin^−/−^ thymii were at least as large as WT thymii and contained at least as many thymocytes (not shown). Flow cytometric analysis showed that T cell development proceeded normally through double negative and double positive stages, and that ezrin^−/−^ thymii ultimately generated normal percentages of CD4 and CD8 single positive T cells (Fig. 7C). Thus, thymic stromal cells deficient for ezrin can support T cell development.

**Figure 8:**
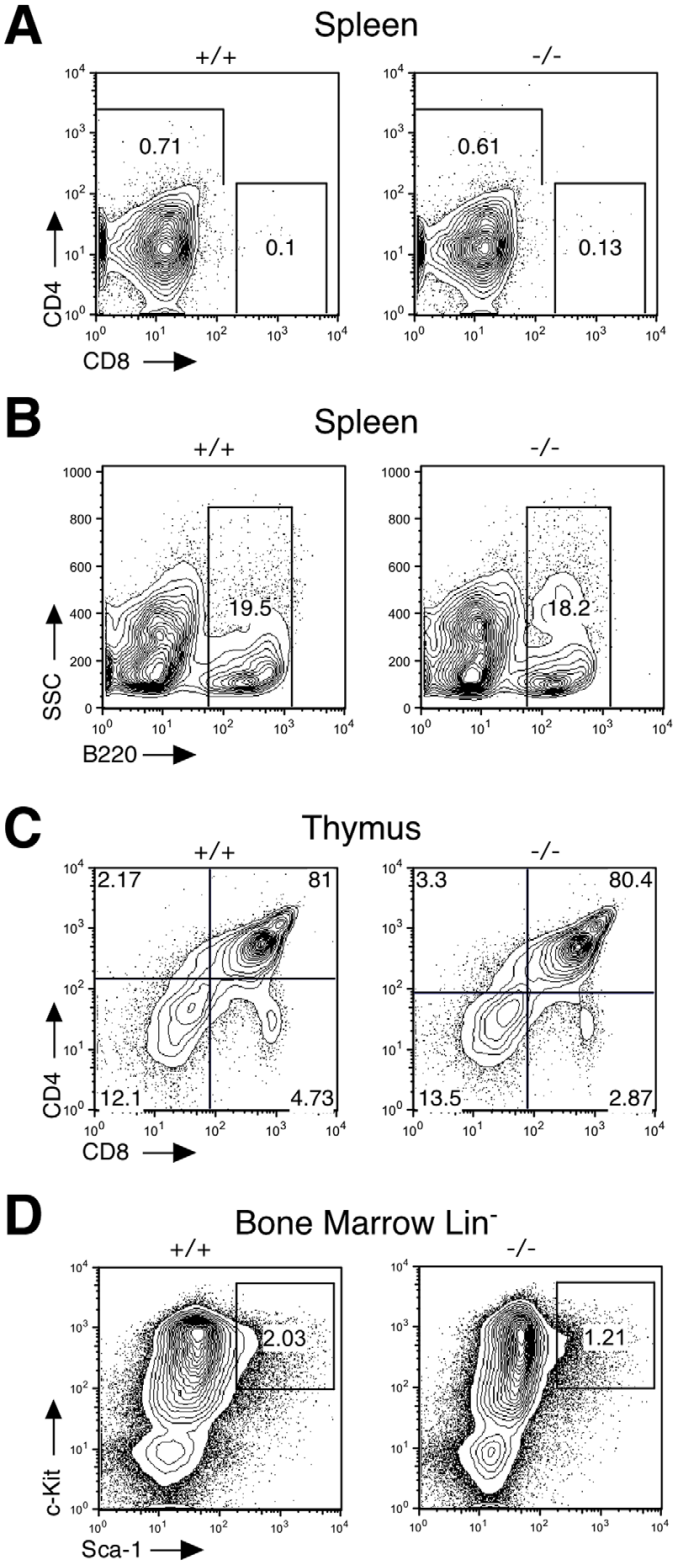
At birth, ezrin^−/−^ mice exhibit normal lymphoid populations. Ezrin^−/−^ mice and wildtype littermates were sacrificed within 12 hours of birth, and single cell suspensions were made from the spleen, thymus, and bone marrow. Splenocytes (A and B), thymocytes (C), or bone marrow cells (D) were stained using antibodies as shown and analyzed by flow cytometry.

Ezrin^−/−^ mice exhibit gut defects [4], raising the possibility that at least some of the defects we observe arise as a secondary consequence of nutritional stress. We therefore analyzed newly-born ezrin^−/−^ mice to ask if the defects in lymphopoiesis are detectable before the onset of nutritional stress. Mice were sacrificed within 0–12 hours of birth, and single cell suspensions from thymus, spleen, and bone marrow were analyzed by flow cytometry. At birth, T cell development is incomplete, and most cells have only reached the double positive stage. Analysis of these mice reflected this; few mature single-positive T cells were present in the spleen or thymus of wildtype or ezrin^−/−^ mice, and the proportion of double positive cells was similar to those found in adult animals (Figure 8A and C). Ezrin^−/−^ mice also showed no defects in the generation of double positive T cells (Figure 8C). Furthermore, no differences were seen in the percentage of B cells in the spleen (Figure 8B) or in hematopoietic precursors in the bone marrow (Figure 8D). Taken together, these findings indicate that ezrin expression is dispensable in hematopoietic and stromal lineages, and that most of the defects in lymphoid development observed in ezrin^−/−^ mice arise as a consequence of nutritional stress.

## Discussion

Recently, it has become clear that ERM proteins play an important role in the function of mature lymphocytes. In this study, we have analyzed for the first time the expression patterns of ERM proteins in lymphoid tissues and conducted the first analysis of the role of ezrin in lymphoid development. We found that ezrin expression is high during early thymocyte development, while moesin expression is low, raising the possibility that ezrin plays a unique role in early thymocyte development. Germline deletion of ezrin results in profound pathology in lymphoid development. However, further analysis showed that most of these defects are stress-induced, presumably arising as a secondary consequence of previously identified defects in the gut. We conclude that ezrin is dispensable for lymphoid development, most likely due to redundancy with moesin.

Our results show that deletion of ezrin in the germline leads to a severe reduction in cellularity of bone marrow, spleen and thymus, and disruption of thymic architecture. Although distinct regions of differentiated cortex and medulla were present in thymii from ezrin^−/−^ mice, severe cortical thinning was observed and medullary organization was abnormal. Acute cortical thinning is commonly caused by increased corticosteroids induced by various stressors, including infection, disease, chemotherapy, irradiation, and malnutrition, or by treatment with dexamethasone (DEX) or 2,3,7,8-tetrachlorodibenzo-p-dioxin (TCDD) [23], [24]. Although the phenotype arising from each of these stressors is slightly different, all result in the reduced thymic output and apoptosis of thymocytes, especially cortical DP T cells [23], [25]. Thus, the defects that we observe in thymocyte number and thymic architecture are consistent with defects seen under stress conditions. Indeed, several pieces of evidence point to a secondary stress response, rather than a direct effect on thymic development. First, changes in thymocyte populations were only observed in mice after several days, by which time runting due to malnutrition was evident. Second, only modest defects were observed in fetal liver chimeras, demonstrating that the requirement for ezrin is independent of hematopoietic-lineage cells. Finally, transplantation of ezrin-deficient thymii into wild type mice also led to normal thymocyte populations, showing that ezrin expression in thymic stromal cells is dispensable for T cell development.

A stress response can also explain the observed defects in splenic lymphocyte populations. The lack of splenic RBCs in ezrin^−/−^ mice suggests the presence of severe anemia. Since no accumulation of dead and dying RBCs in the spleens of ezrin^−/−^ mice was observed, it is likely that these mice fail to generate sufficient quantities of mature RBCs. Although measurements of the hematocrit from ezrin^−/−^ mice were not consistent with anemia, inaccurate hematocrit and hemoglobin counts could be caused by dehydration. Dehydration has not been directly documented in ezrin^−/−^ mice, but ezrin has been shown to affect the function of the sodium-hydrogen exchanger (NHE3), which is required for fluid homeostasis in the gut and renal proximal tubule [24], [26]. Importantly, the red cell distribution width (RDW) of RBCs was increased in ezrin^−/−^ mice, suggesting that the time of erythropoiesis was shortened to counteract anemia. In humans, the most common causes of decreased mean corpuscular hemoglobin concentration (MCHC) and increased RDW are iron or vitamin deficiencies [27]. Therefore, it seems likely that the blood abnormalities we observe are attributable to nutritional deficiencies and dehydration. In support of this view, analysis of RBC development in bone marrow of ezrin^−/−FLCh^ mice showed no defects, and RBCs efficiently entered the spleen in these mice (data not shown).

While most of the immunologic defects that we observed in ezrin^−/−^ mice are almost certainly a secondary consequence of defects in other organ systems, we noticed mild cortical thinning in both T cell conditional knockout mice and fetal liver chimeras (MHS, unpublished observations), suggesting that ezrin expression in thymocytes may play a small role in thymic development. Thymic cellularity was normal in both model systems, arguing against enhanced apoptosis of thymocytes. Since mature conditional ezrin^−/−^ T cells exhibit defects in migration and adhesion (MHS and JKB, manuscript in preparation), the observed cortical thinning may be associated with improper movement of ezrin^−/−^ thymocytes within the thymus.

Although we found that ezrin is dispensable for lymphoid development, we found an intriguing pattern of ERM protein expression in developing thymocytes. Mature lymphocytes and peripheral lymphoid organs show a characteristic pattern of ERM protein expression, with moesin expressed at three fold higher levels than ezrin, and little to no radixin expressed [28]. In contrast, we find that ezrin expression is higher in the thymus, where it is roughly equimolar with moesin. Real-time PCR analysis showed that ezrin and moesin are differentially regulated during development, with ezrin expression highest in early thymocytes, and moesin highest in mature thymocytes and peripheral T cells. Assuming that expression of these proteins is controlled at the mRNA level, then ezrin is expressed at levels equal to or higher than moesin in early thymocytes, and the 3∶1 moesin∶ezrin pattern characteristic of mature T cells emerges late in development. In keeping with this, our histological analysis shows strong ezrin expression in the thymic cortex, where early T cell development takes place, and strong moesin expression in the medulla, which is enriched in more mature T cells. Interestingly, ezrin mRNA levels diminish sharply between the DN3 stage and the ISP stage of development, at the time when the pre-TCR is expressed and positive and negative selection occurs. It is also at this stage of development that moesin levels begin to rise. This raises the possibility that the switch in ERM protein expression is triggered by pre-TCR signaling and thymic selection.

The significance of these expression patterns is currently unclear. Indeed, the general significance of tissue-specific expression of ERM proteins is unknown. Although distinct expression patterns may have arisen as a result of gene duplication and random genetic drift, it is appealing to speculate that these proteins serve overlapping, but distinct functions. In support of this idea, ezrin and moesin show distinct patterns of movement and post-translational modification during T cell activation [12], [28], [29] and proteins such as L-selectin and CD95/Fas bind ezrin and moesin differentially [9], [18]. Thus, these differences could make ezrin best suited to support early thymocyte development, and moesin better suited to support mature T cell function. Even if this is the case, however, our analysis of ezrin^−/−^ mice indicates that thymic development can proceed in the absence of ezrin expression, as long as nutritional stress is circumvented. Moesin mRNA is upregulated in ezrin^−/−^ thymii, and likely also undergoes hyperphosphorylation similar to that we have observed in mature ezrin^−/−^ T cells [12]. It seems likely that moesin can compensate for loss of ezrin, though this will need to be formally tested by generation of double knockout mice.

## Materials and Methods

### Ethics Statement

All animal studies were carried out according to guidelines put forth by the NIH guide for the care and use of laboratory animals, as approved under protocol #2008-10-667 by the Children's Hospital of Philadelphia Institutional Animal Care and Use Committee.

### Mice

To generate ezrin^−/−^ mice, mice homozygous for a floxed ezrin gene (ezrin^fl/fl^) [4], were crossed with EIIA-Cre transgenic mice [30] to generate mice with deletion of ezrin early in embryogenesis. F1 chimeras were genotyped for both EIIA-Cre and the presence of an excision event, and then crossed an additional time to ezrin^fl/fl^ mice. These mice were genotyped for both the null allele and the absence of the Cre transgene, an outcome which indicates the null allele was present in the gamete. Since this created germline deletion of ezrin, these mice are hereby referred to as ezrin^+/−^ mice. Heterozygous mice were maintained by crossing to C57Bl/6 mice. Wildtype littermates were used as controls.

### Fetal liver chimeras

Fetal livers were collected at E14 from ezrin^+/−^ pregnant mothers mated to ezrin^+/−^ males. Additional tail tissue was also collected for genotyping. Single cell suspensions were generated, and liver cells were pelleted by centrifugation, resuspended in 200µl freezing media (10% DMSO in 90% FBS), and frozen at −80°C. To generate chimeric animals, wildtype congenic (CD45.1) recipient mice were lethally irradiated with 800 rads, followed two hours later with a second dose of 400 rads. Fetal liver cells (about ¼ of total cells from 1 liver) were injected into the tail vein of recipient mice. Mice were then allowed to recover for 6–8 weeks, and kept on either a chlorinated or antibiotic-containing water source.

### Thymic Transplantation

Subcutaneous thymic transplants were performed based on the procedure of Miller [31]. Briefly, donor thymii were obtained from one to three day old ezrin^−/−^ pups or littermate controls and placed into ice-cold, sterile PBS. Wildtype C57Bl/6 mice were used as recipients. A lateral incision was made at the mid-axillary line at the 4th intercostal space and following subcutaneous dissection, the thymus was placed into the axilla. The wound was covered with topical antibiotics (Bacitracin, Clay-park Labs, Bronx, NY) and closed with nylon sutures. Mice were euthanized after nine weeks, and organs were harvested for analysis.

### Hematology, Flow Cytometry and cell sorting

Blood was collected by cardiac puncture and analyzed using a Hemavet 950. For flow cytometry, single cell suspensions from the indicated tissues were stained with the following fluorescently-conjugated monoclonal antibodies: CD3-PE, CD4-APC, CD4-FITC, CD8-PECy7, CD8-APC, CD25-PE, CD69-PE, B220-PE, CD62L-PE, CD44-FITC, or IgM-FITC (all from Biolegend). Antibodies specific for Sca-1, c-kit, and a hematopoetic linage cocktail (lin1) were obtained from Becton-Dickinson. Flow cytometry was performed using FACS Caliber, LSRII, or FACS Canto (Becton-Dickinson) flow cytometers, and analysis was done using FlowJo (Treestar).

Antibodies used for sorting of thymic subsets (below) include: G8.8-AF647, CD11c (Biolegend); HSA-FITC CD8-PerCPCy5.5, TCRβ-PE, CD44-APC, CD25-APC Cy7 (Becton-Dickinson); CD45.1-PE Cy7, CD19, NK1.1, Gr-1, CD4-PE Cy7 (E-biosciences); 4/80, B220, CD11b (Caltag); and Live/Dead Aqua (Invitrogen).

### Sorting of thymic subsets and real time PCR

Thymic subsets were sorted based on live/dead and expression of cell surface markers. Thymic epithelial cells were isolated by incubating thymi with mixture of Collagenase A (Roche) and DNAse (Sigma) for 30 minutes at 37°C. Epithelial cells were then separated by Percoll gradient centrifugation followed by sorting for live/dead and expression of Ep-CAM and lack of Ly5. RNA was isolated with Trizol LS Reagent following the manufacturers instructions (Invitrogen), and used to generate cDNA using SuperScriptII Reverse Transcriptase and random hexamer primers (Invitrogen). RT-PCR was performed using TaqMan Fast Universal Mastermix (Applied Biosystems) in a total volume of 10ul on an ABI 7000 Sequence Detection System. Primers used included ezrin, radixin, moesin, and beta-actin (Applied Biosystems: Mm00447761_m1, Mm00501337_m1, Mm00447889_m1, and Mm00607939_s1 respectively). Transcript abundance was normalized to expression of beta-actin within each sample, and relative expression was compared with that of whole thymus.

### Histology

Histological analysis was performed by the The Children's Hospital of Philadelphia Pathology Core or the University of Chicago Immunohistochemistry Facility. Briefly, thymii and spleens were fixed in 4% formalin and imbedded in paraffin for staining with hematoxylin and eosin. Alternatively, samples were frozen in OCT compound (Tissue Tek), and frozen sections were labeled with anti-K5 (AF138, Covance), anti-K8 (Troma-I, Developmental Studies Hybridoma Bank), anti-CD11b, anti-Gr-1 (ebioscience) or *Ulex Europaeus* Agglutinin I (UAE, Vector Laboratories).

### Western Blots

Lymphoid tissues were either lysed directly in SDS sample buffer using a dounce homogenizer flowed by boiling, or snap frozen and homogenized prior to resuspension in lysis buffer (1% Triton X-100, 150mM NaCl_2_, 5mM EDTA, 50mM Tris-HCl, pH7.5, with protease, phosphatase and kinase inhibitors). Following separation by SDS-PAGE electrophoresis (Tris-glycine, 10% acrylamide), proteins were transferred to nitrocellulose and blocked in 3% BSA in PBS. Blots were probed with antibodies specific for rabbit anti-phospho-ERM (Cell Signaling), mouse mAb 3C12 anti-ezrin (Neomarkers), or mouse mAb anti-GAPDH (Millipore), followed by IR800 goat anti-rabbit Ig, (Rockland) or AlexaFluor680 donkey anti-mouse Ig (Invitrogen). Blots were visualized using the Odyssey Imager (Licor), and quantitation was performed within the linear range.
